# Development of a Parental Feeding Goal Measure: The Family Mealtime Goals Questionnaire

**DOI:** 10.3389/fpsyg.2019.00455

**Published:** 2019-03-12

**Authors:** Sarah Snuggs, Carmel Houston-Price, Kate Harvey

**Affiliations:** School of Psychology and Clinical Language Sciences, University of Reading, Reading, United Kingdom

**Keywords:** goals, priorities, eating behavior, family, mealtimes, surveys and questionnaires

## Abstract

It is well established that parents' feeding practices predict children's eating behaviors. However, there has been little research into parents' mealtime goals—their desired outcomes for family mealtimes. These goals, and potential conflicts between them, may be important both in explaining parents' feeding practices and improving children's eating behaviors, as health behavior change is more likely to be achieved by programmes and interventions that are aligned with an individual's goals. The objectives of this study were to develop a reliable and valid measure that captures parental mealtime goals, and to describe parents' endorsement of these goals. Online questionnaire methods were used to design and test the Family Mealtime Goals Questionnaire with 1,140 parents and carers of at least one child aged from 1 to 16 years. Exploratory qualitative analysis, Principal Components Analysis, Confirmatory Factor Analysis, and test-retest analysis (using intraclass correlations) were conducted to establish the psychometric properties of the instrument. An 18-item questionnaire was produced with seven dimensions: *stress/conflict avoidance, homemade food, shared family food, family involvement in mealtimes, price, occasional treats*, and *high/low fat regulation*. Some differences were found in the goal structure of parents of children of different ages but *stress/conflict avoidance* was the most strongly endorsed mealtime goal for all age groups. The Family Mealtime Goals Questionnaire provides a useful measure of parents' feeding motivations. It will facilitate large-scale research into the relationships between parents' feeding goals and practices and could inform the design of more effective healthy eating interventions that target specific feeding goals.

## Introduction

Research has established that parents' feeding practices predict children's eating behaviors (Patrick and Nicklas, 2005; Pearson et al., 2009; Carnell et al., 2014). Possible mechanisms for this influence include parenting style, modeling of eating behavior, family meal frequency and exposure to food, all of which are associated with child eating behaviors, such as fruit and vegetable intake (Birch, 1999; Birch et al., 2007; Mitchell et al., 2013). One further potential factor, which has been little explored to date, is parents' mealtime goals or parents' desired outcomes for family mealtimes. Individual goals (defined as “internal representations of a desired state”) (Austin and Vancouver, 1996) are known to predict health-related behaviors, such as dieting and physical activity (Presseau et al., 2010; Papies, 2012). Moreover, interventions tailored to an individual's goals are more successful in invoking health behavior changes (Papies, 2012). However, goal attainment is found to be hindered if goals are perceived to conflict with one another (Emmons and King, 1988; Boudreaux and Ozer, 2013). One might therefore expect parents' mealtime goals—and any conflicts between these—to play an important role in determining their feeding behaviors. However, there has been surprisingly little research into the nature of parent's mealtime goals, or how these might be harnessed to support healthy eating interventions. This study set out to develop a tool that could be used for this purpose.

Family mealtimes with positive family dynamics play an important role in children's healthy eating (Hammons and Fiese, 2011; Dwyer et al., 2015). More frequent shared family meals predict greater intake of fruit, vegetables, and key nutrients such as fiber, calcium, and iron (Gillman et al., 2000) and might be protective against obesity and disordered eating (Ackard and Neumark-Sztainer, 2001; Berge et al., 2014) and support general emotional wellbeing (Utter et al., 2017). The mechanisms underlying this relationship are unclear but may include lower reliance on pre-packaged food (and therefore more exposure to home-made foods) when families eat together, along with greater opportunities for parents to model healthy eating behaviors and notice when children are eating unhealthily (Gillman et al., 2000; Fulkerson et al., 2006; Hammons and Fiese, 2011). Parents' goals may differ when planning shared family meals vs. providing other kinds of meals or snacks. Yet, few studies have examined parents' motivations when choosing foods for children to eat alone or with the family, and none have examined their goals specifically in relation to mealtimes.

Recent qualitative studies suggest that, while health is a key motivator of parents' food choices for children, practicality, cost, appetite management and weight control are also important (Moore et al., 2010; Carnell et al., 2011). Furthermore, St John Alderson and Ogden (St John Alderson and Ogden, 1999) found that parents fed their children fewer healthy foods than they ate themselves, despite placing more emphasis on health when describing their motivations for selecting their children's food. This suggests that, although parents hold a health goal for their children's meals, other goals are prioritized during mealtime decision-making. Additionally, how parents interpret “health” and “convenience” in relation to their mealtime goals remains unclear, and this might differ between individuals. For example, when thinking about the importance of providing healthy meals for their children, some parents might consider the nutritional quality of the child's dietary intake whilst others may be more concerned with establishing healthy eating behaviors, such as shared family meals.

Previous investigations of the relationship between parents' feeding goals, feeding practices and children's eating behaviors (Roos et al., 2012; Kiefner-Burmeister et al., 2014; Russell et al., 2015; Hoffmann et al., 2016) have typically used adaptations of the Food Choice Questionnaire (FCQ) (Steptoe et al., 1995), originally designed to measure adults' reasons for their own food choices. This work suggests that parents' health-related goals are associated with positive eating behaviors among children, while “convenience” goals predict both negative feeding practices (e.g., using food as a reward, feeding for emotion regulation) and negative eating behaviors (e.g., candy consumption). However, synthesizing the findings of these studies is hindered by their use of differing versions of the questionnaire. For example, while Russell et al. (2015) found the parental goal of “giving the child what s/he wants” to predict low liking of vegetables by children, this goal was not assessed in other studies.

While the original FCQ has good psychometric properties, the reliability and validity of the instruments adapted to explore parents' motivations were examined in only two of the above studies (Roos et al., 2012; Russell et al., 2015). Most factors demonstrated good Cronbach's α values and factor loadings, but several were lower than those reported for the original FCQ. Of greater concern are the face and content validity of the adapted questionnaires, which focus on *Convenience* and *Health* factors, and may not fully capture parents' goals when making decisions about the food they provide for their children. For example, recent qualitative work investigating reasons for feeding children pre-packaged food identified lack of time, meal-planning ability and family preferences as key motivations (Horning et al., 2017), only some of which are captured by the items in the FCQ (e.g., no items measure the influence of family preferences). The FCQ items for the “health” and “convenience” factors are also insufficiently specific to elucidate parents' interpretations of these terms, and whether these differ between parents, especially in the case of health goals (e.g., “It is important to me that the food my child eats keeps him/her healthy” does not reveal parents' understanding of the concept of “health”). Finally, the FCQ primarily measures the reasons behind parents' selection of foods for their child, rather than their mealtime goals per se.

We therefore set out to develop a tool that would more directly assess and operationalize parents' goals when planning and making decisions about mealtimes. Preliminary research explored parents' understanding of an adapted FCQ using a “think aloud” technique (Ericsson and Simon, 1998); this revealed that, while several of the factors measured by the FCQ aligned with parents' broad motivations in relation to mealtimes (e.g., health, price, convenience), some items lacked face validity (Snuggs et al., 2016). In line with St John Alderson and Ogden (St John Alderson and Ogden, 1999), we found that parents' motivations differ substantially when choosing foods for themselves vs. for their children, suggesting that goals for children's mealtimes are likely to be distinct from parents' own food choice goals [e.g., whether to involve children in food decisions (Carnell et al., 2011)]. This preliminary research led us to conclude that a new measure was required to capture parents' goals and priorities when feeding their children. Such a measure could be used not only to describe parents' feeding goals but also to establish how these goals, and any conflicts between them, influence parents' feeding behaviors and children's eating behaviors in both the general population and in cases of pediatric feeding/eating disorders. As a first step, the objectives of the current study were to develop a reliable and valid measure for this purpose and to describe the mealtime goals endorsed by parents using this instrument.

## Development of the Family Mealtime Goals Questionnaire

The questionnaire was developed and tested in three stages. First, a large item pool was generated in order to capture as many potential goals as possible; this was then refined using qualitative methodology to produce a preliminary questionnaire. Next, the preliminary questionnaire was administered to an initial sample of parents, after which Exploratory Factor Analysis methods were used to produce a provisional questionnaire. This was subjected to Confirmatory Factor Analysis and test-retest analysis in a new sample of parents. We describe the procedure followed and the results of this stage-by-stage below.

All parts of the study were granted approval to proceed by the University of Reading Research Ethics Committee.

### Development of the Item Pool

#### Methods

A systematic approach to measure development (Churchill, 1979; Clark and Watson, 1995) was followed to capture as many potential goals as possible. An initial pool of items was produced from three sources:

1. Parents who responded to social media posts placed on several web-based parenting forums (*N* = 61). Members of these forums were invited to provide written responses to the open-ended question, “What is your goal when providing a meal for your child?”
2. Secondary analyses of data from parents who had participated in an earlier, unpublished study involving a family eating intervention (*N* = 990). When asked to provide written responses to an open-ended question about what they hoped to gain from the intervention, parents often mentioned mealtime goals; these responses were included in the item pool.
3. Items used in previous studies of food choice motivation (e.g., items reflecting factors such as *Convenience* from the FCQ) and feeding practices [e.g., introducing unfamiliar foods Musher-Eizenman and Holub, 2007]; practicality and appetite management (Moore et al., 2010; Carnell et al., 2011).

Finally, we cross-checked and confirmed that the proposed motivators identified by the literature mentioned in the Introduction were covered by the items generated from these sources.

#### Results

The development process provided 130 items (113 after de-duplication) describing the feeding goals of parents from a wide range of socio-economic backgrounds and age groups. As recommended by DeVellis (DeVellis, 2017), these items were sent to a group of expert academics (developmental psychologists and nutritionists) all of whom were also parents (*n* = 8). The experts were asked to highlight any items that were ambiguous or difficult to answer (to ensure face validity), and to identify any goals that were missing from the list of items (to ensure content validity). Based on the expert feedback and item-formatting guidance (Dolnicar, 2013; DeVellis, 2017), we adjusted or removed duplicate or confusing items. Experts did not identify any missing goals. This resulted in a preliminary Mealtime Goal questionnaire containing 66 items presented in a random order on a 5-point Likert scale (see Appendix I).

### Testing of the Preliminary Questionnaire

#### Methods

The preliminary questionnaire was administered to a pool of parents to allow exploratory analysis to identify the components underlying their responses. For this purpose, we used Principal Components Analysis (PCA), commonly used for exploratory analysis in scale development (Hinkin et al., 1997; DeVellis, 2017).

##### Participants

Parents (*N* = 515) were recruited through online social media platforms, including national parenting forums and regional family websites, and through snowball sampling (participants were encouraged to share the questionnaire link with friends and family). Participants were excluded if they stated that no children lived with them some or all of the time. Participants with more than one child were asked to answer questions in relation to the child whose name began with the letter closest to the beginning of the alphabet. To help ensure consistency during completion, parents provided the name of the child they were answering in relation to and this appeared continuously on the screen as a prompt. Several participants failed to provide socio-demographic information, but all participants who completed the goal questionnaire in full (*N* = 407) were included in analyses. A description of the sample of parents who completed the preliminary questionnaire is provided in Table 1.

**Table 1:** Participant characteristics: testing of preliminary questionnaire using PCA.

	**Mean**	**Std. deviation**
Number of children living at home	1.6	0.8
Child age (*n* = 398)	4.6	3.7
Parent age (*n* = 404)	37.1	6.5
	**%**	***n***
% female (child)	48.2	196
% female (participant) (*n* = 388)	91.7%	356
**RELATIONSHIP TO CHILD**		
Parent/step-parent	97.3	396
Grandparent	1.2	5
Other	1.5	6
**PARTICIPANT ETHNIC ORIGIN**		
White-British	76.2	310
White-Other	17.2	70
Other	4.2	17
Not stated	2.5	10
**PARTICIPANT OCCUPATION**		
In employment	72.4	295
Stay at home parent	16.5	67
Student	1.2	5
Other	9.8	40
**PARTICIPANT EDUCATION LEVEL**		
Undergraduate degree or higher	78.4	319
Post-secondary/vocational qualification	12.5	51
Secondary education	4.9	20
Did not complete secondary education/other	4.2	17

*PCA, Principal Components Analysis. N = 407 unless otherwise stated (ns < 407 indicate missing data)*.

##### Procedure

The 66-item preliminary Mealtime Goal questionnaire was scripted onto an online survey platform^1^ and a link to the survey was posted on relevant parenting sites. Participants were asked, “Thinking about your child's mealtimes, how strongly do you agree with the following statements?” Participants were asked to provide socio-demographic information (OfNS, 2005; Sapsford, 2007; Connelly et al., 2016) and to rate their agreement with each statement about their mealtime goals on a 5-point scale (Strongly agree, Agree, Neither agree nor disagree, Disagree, Strongly disagree). Items were presented in random order. In line with recommendations that scale development requires 5–10 participants per item and a total sample of at least 150 (Hinkin et al., 1997), the questionnaire remained available until the sample exceeded 400.

#### Results

The data were screened for their suitability for Principal Components Analysis (PCA). First, parents' responses to each item were examined and those with severely skewed distributions (i.e., eliciting agreement or disagreement by >98% participants; *n* = 21) were discarded.

Responses to items relating to general “health” were heavily skewed at the preliminary PCA stage, due to all parents endorsing them, and so are not included in Table 2. However, due to the potential importance of this factor (and of the conflict between health-related and other goals), 3 health-related items were retained at this stage, with a view to exploring further at the CFA stage (*Optional Component 9* in Appendix III). Correlations were computed between responses to each item; none exceeded 0.9, the value used to indicate that multi-collinearity is present (Hair et al., 2010).

**Table 2:** PCA component loadings and Cronbach's α values: preliminary questionnaire.

	**Component loading**	**Cronbach's α**
**COMPONENT 1: SHARED FAMILY FOOD**		
I don't want to prepare different foods for different family members	0.796	0.723
I want my child and me to eat the same food	0.794	
I want to prepare food that all my family will eat	0.754	
**COMPONENT 2: STRESS/CONFLICT AVOIDANCE**		
I want to avoid arguments at mealtimes	0.781	0.691
I don't want to get stressed thinking about mealtimes	0.763	
I want to make sure I don't lose my temper at mealtimes	0.759	
**COMPONENT 3: HOMEMADE FOOD**		
I want to prepare food for my child using natural ingredients	0.784	0.669
I want to prepare food for my child using raw ingredients	0.743	
I want to give my child home-cooked food	0.727	
**COMPONENT 4: FAMILY INVOLVEMENT IN MEALTIMES**		
I want the whole family to help out with mealtimes	0.816	0.671
I want to choose food that my child can help prepare	0.708	
I want to get my child involved with things like setting the table or clearing up	0.669	
**COMPONENT 5: EASE OF PREPARATION**		
I want to choose food for my child that is easy for me to prepare	0.878	0.748
I don't want to spend a long time preparing food for my child	0.875	
**COMPONENT 6: PRICE**		
I want to keep to my budget	0.887	0.767
I want to keep costs down	0.842	
**COMPONENT 7: OCCASIONAL TREATS**		
I want to give my child sugary treats sometimes	0.856	0.661
I want my child to be free to eat unhealthy food sometimes	0.845	
**COMPONENT 8: HIGH AND LOW FAT REGULATION**		
I want to give my child food that is low in fat	0.837	0.581
I don't want to give my child fatty foods	0.815	

A PCA was carried out on the remaining 45 items using SPSS version 24, adopting standard procedures and thresholds unless otherwise stated. Varimax rotation with Kaiser normalization was used, applying the Kaiser criterion (Eigenvalue >1) (Kaiser, 1960) and suppressing loadings <0.4 (Hinkin et al., 1997). The first PCA resulted in nine items being dropped due to loading onto more than one component (Hair et al., 2010). The PCA was repeated, identifying 10 components comprising 30 items (six items with a factor loading <0.4 were suppressed). Inter-item correlations were computed within components; two components (containing 10 items) with *r* values substantially below 0.4 were discarded (Hinkin et al., 1997).

Inter-item reliability was measured for the eight remaining components and all but one had Cronbach's α >0.65 suggesting medium to good reliability (see Table 2). The remaining component (α = 0.581) was retained for the next stage of analysis with a view to discarding it if it remained unreliable. The Kaiser Meyer Olkin Index for the model containing 8 components was 0.714 (*p* < 0.001), indicating adequate sampling.

Further PCA analyses were run to explore the responses of parents of younger and older children (split by median child age) separately. In both samples, the components shown in Table 2 were broadly supported by both PCA and Maximum Likelihood Exploratory Factor Analysis (Kaiser Meyer Olkin Index = 0.72 and 0.62 for the younger and older samples respectively).

The 20 items shown in Table 2 were therefore retained in the Family Mealtime Goals Questionnaire, to be tested in a confirmatory stage of analysis involving a separate sample of parents.

### Testing the Family Mealtime Goals Questionnaire

#### Methods

The Family Mealtime Goals Questionnaire was administered to a new sample of parents, and Confirmatory Factor Analysis (CFA) was used to verify the factor structure of the model described above. A subset of the data were also used to establish test-retest reliability of the new measure.

##### Participants

A new sample of parents were recruited to complete the questionnaire through adverts on parenting websites and snowball sampling, with the same exclusion criteria as the previous stage. All participants who completed the goal questionnaire in full (*n* = 733) were included in analyses. Participant characteristics are described in Table 3.

**Table 3:** Participant characteristics: testing the provisional family meatime goals questionnaire using CFA.

	**All participants**		**Participants who contributed to Reliability testing**	
	**Mean**	**Std. deviation**	**Mean**	**Std. deviation**
Number of children living at home	1.8	0.81	1.8	0.9
Child age (*n* = 729)	6	3.87	6.3	5.1
Parent age (*n* = 729)	37.8	7.03	37.1	7.6
	**%**	***n***	**%**	***n***
% female (child) (*n* = 728)	50.8	372	47.3	87
% female (participant) (*n* = 746)	96.9	723	94.9	168
**RELATIONSHIP TO CHILD (*****n*** **=** **729)**				
Parent/step-parent	99	723	99.5	184
Grandparent	0.5	4	0.5	1
Other	0.2	2		0
**PARTICIPANT ETHNIC ORIGIN**				
White-British	73.2	537	67.6	127
White-Other	11.7	86	22.3	42
Other	13.1	96	5.9	11
Not stated	1.9	14	4.3	8
**OCCUPATION (*****n*** **=** **718)**				
In employment	65.2	468	64.6	115
Stay at home parent	23.8	171	25.3	45
Student	2.4	17	2.3	4
Other	8.6	62	7.9	14
**PARTICIPANT EDUCATION LEVEL (*****n*** **=** **692)**				
Undergraduate degree or higher	61.2	424	62	106
Post-secondary/vocational qualification	28.3	196	29.2	50
Secondary education	8.7	60	7	12
Did not complete secondary education/other	1.7	12	1.8	3

*CFA, Confirmatory Factor Analysis. For CFA, N = 733 unless otherwise stated (total ns < 733 indicate missing data). For test-retest, n = 188 unless otherwise stated (total ns < 188 indicate missing data)*.

##### Procedure

The Family Mealtime Goals Questionnaire, consisting of the 20 items in Table 2, was scripted onto the same online survey platform as before with the same instructions, randomizing order of item presentation.

#### Results

**Figure 1:**
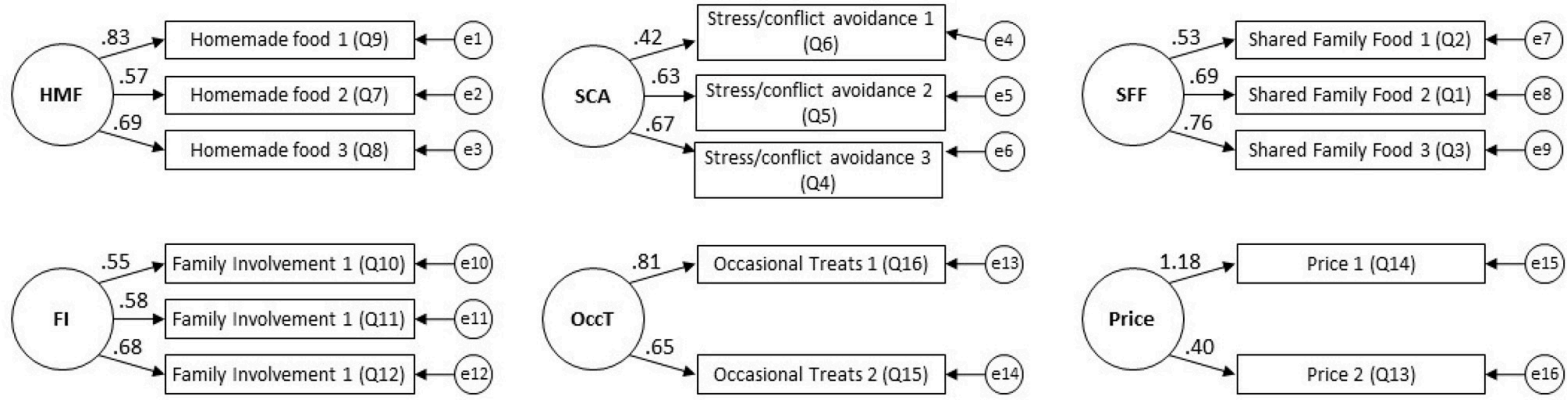
Summary of confirmatory factor analysis model.

Confirmatory Factor Analysis (CFA) was conducted using AMOS version 24. The a-priori 8-factor model identified by the PCA did not provide a good fit for the data from the second cohort of parents. However, when the *ease of preparation* factor was removed, the model was supported (χ^2^ = 275.07, *df* = 114, *p* < 0.01. RMSEA = 0.04 (90% CI [0.037, 0.051]), CFI = 0.95, TLI = 0.93). See Figure 1 for the loading of each item in the final model. Cronbach's α for each factor is provided in Table 4; all values >0.6. The aforementioned component with a lower alpha value in the PCA stage had a notably higher value in the CFA stage (0.74) and its items were therefore retained in the final questionnaire. The three “health” items mentioned in the PCA stage were, again, almost universally endorsed and also worsened the CFA model fit. They were therefore discarded at this point, although remain in Appendix III for transparency and further research.

**Table 4:** Cronbach's α values from CFA (testing provisional family mealtime goals questionnaire).

	**α**
Shared family food	0.73
Stress/conflict avoidance	0.62
Homemade food	0.71
Family involvement in mealtimes	0.65
Price	0.78
Occasional treats	0.67
High and low fat regulation	0.72
(Ease of preparation	0.74)

*CFA, Confirmatory Factor Analysis*.

To investigate whether parents' goals for children's mealtimes change as children age, separate Confirmatory Factor Analyses were conducted for three age groups: 1–3 year-olds (pre-schoolers), 4–10 year-olds (primary school children) and 11–16 year-olds (secondary school children). Considerable overlap can be seen between the models, indicating that goals are very similar, but not identical, for parents of different age groups (see Appendix II).

Model fit for the pre-schooler group (*n* = 258) was best when both the *ease of preparation* and *high & low fat regulation* factors were dropped (χ^2^ = 163.760, *df* = 89, *p* < 0.01, RMSEA = 0.06 (90% CI [0.03, 0.09]), CFI = 0.91, TLI = 0.88). The best model fit for the primary school group (n = 390) was the same 7-component model that best fitted the whole sample (i.e., dropping the *ease of preparation* factor) (χ^2^ = 186.609, *df* = 114, *p* < 0.05. RMSEA = 0.04 (90% CI [0.030, 0.051]), CFI = 0.96, TLI = 0.95). The secondary school group was a relatively small sample (*n* = 115), with participant numbers falling below scale development recommendations (Hinkin et al., 1997). Consequently, results should be treated with caution, as emphasized by the confidence interval data. The model best supported by the data for this age group was the original model identified at the PCA stage (i.e., including *ease of preparation*), but with *occasional treats* removed, along with one further item (χ^2^ = 118.928, *df* = 98, *p* = 0.074. RMSEA = 0.04 (90% CI [0.00, 0.69]), CFI = 0.96, TLI = 0.95) (See Appendix II).

The final questionnaire therefore includes the 7 components and 18 items confirmed to provide the best fit for the cohort as a whole (see Appendix III). The questionnaire assesses parents' goals in relation to the following factors: *shared family food; stress/conflict avoidance; homemade food; family involvement in mealtimes; price; occasional treats; high and low fat regulation*. Items relating to the additional component of “*ease of preparation*” are also provided, to enable the tool's use by parents of older children, as are items relating to a global “*health”* component, which did not meet criteria for retention but may be of interest in further research.

### Test-Retest Reliability

#### Methods

An opportunity sample of participants at this stage (*n* = 303) were recruited specifically to participate in test-retest analyses. These participants displayed similar characteristics to the rest of the sample (see Table 3) and were sent an email containing a link to an identical questionnaire 1 week after completion of the first questionnaire, with a follow-up reminder 1 week later if necessary. All participants who completed the second questionnaire were included in test-retest reliability analyses (*n* = 188, 62%).

#### Results

Table 3 shows the characteristics of the participants who also completed the second questionnaire.

Table 5 shows median scores for this sub-group at both time points and the intra class correlation coefficients between these (Weir, 2005). A 2-way mixed effects model was applied, as recommended (Koo and Li, 2016). All correlations were significant at the 0.001 level and values ranged from 0.48 to 0.8, indicating some temporal variability.

**Table 5:** Test-retest reliability of provisional family mealtime goals questionnaire.

	**Time 1 Median**	**Time 1 IQR**	**Time 2 Median**	**Time 2 IQR**	**ICC**
Shared family food	4.33	0.67	4.33	0.67	0.66^*^
Stress/conflict avoidance	4.33	1	4.33	1	0.48^*^
Homemade food	4	1	4	1	0.66^*^
Family involvement in mealtimes	4	0.67	4	0.67	0.65^*^
Price	4	1	4	1	0.66^*^
Occasional treats	4	0	4	0.5	0.67^*^
High and low fat regulation	3	1.5	3	1.5	0.80^*^
(Ease of preparation)	3.5	1	4	1	0.65^*^

* *p < 0.001. IQR, Interquartile range; ICC, Intraclass Correlation*.

## Parents' Endorsement of Feeding Goals

Data from both samples of parents (those who completed either the preliminary or final questionnaires) were combined to examine parents' endorsement of the measure's goals. Table 6 shows the scores for each mealtime goal both for the whole sample (*N* = 1,140) and by age group. Scores were calculated by taking the mean item score for each component (there are no reversed items).

**Table 6:** Endorsement of feeding goals.

	**Mean**	**Median**	**SD**	**Minimum**	**Maximum**
**WHOLE SAMPLE (*****n*** **=** 1,140^*^)					
Stress/conflict avoidance	4.30	4.33	0.55	2.00	5.00
Homemade food	4.22	4.33	0.58	1.00	5.00
Shared family food	4.21	4.33	0.69	1.00	5.00
Family involvement in mealtimes	3.95	4.00	0.61	1.33	5.00
Price	3.91	4.00	0.77	1.00	5.00
Occasional treats	3.83	4.00	0.63	1.00	5.00
High and low fat regulation	3.27	3.00	0.92	1.00	5.00
**PARENTS OF PRE-SCHOOLERS (< 4 years) (*****n*** **=** **455)**					
Stress/conflict avoidance	4.29	4.33	0.56	2.00	5.00
Homemade food	4.18	4.00	0.61	1.00	5.00
Shared family food	4.17	4.33	0.71	2.00	5.00
Family involvement in mealtimes	3.90	4.00	0.65	1.33	5.00
Price	3.85	4.00	0.79	1.00	5.00
Occasional treats	3.72	4.00	0.73	1.00	5.00
(High and low fat regulation	3.06	3.00	0.93	1.00	5.00)
**PARENTS OF PRIMARY SCHOOL AGED CHILDREN (4–10 years) (*****n*** **=** **517)**					
Stress/conflict avoidance	4.31	4.33	0.56	2.33	5.00
Shared family food	4.25	4.33	0.69	1.00	5.00
Homemade food	4.24	4.33	0.57	2.33	5.00
Family involvement in mealtimes	3.99	4.00	0.58	2.33	5.00
Price	3.97	4.00	0.77	1.00	5.00
Occasional Treats	3.91	4.00	0.54	1.50	5.00
High and low fat regulation	3.40	3.50	0.86	1.00	5.00
**PARENTS OF SECONDARY SCHOOL AGED CHILDREN (11–16 years) (*****n*** **=** **159)**					
Stress/conflict avoidance	4.30	4.33	0.48	2.67	5.00
Shared family food	4.25	4.33	0.66	2.33	5.00
Homemade food	4.23	4.00	0.49	3.00	5.00
Family involvement in mealtimes	4.00	4.00	0.59	2.67	5.00
Price	3.91	4.00	0.74	2.00	5.00
(Occasional treats	3.89	4.00	0.53	2.00	5.00)
Ease of preparation	3.56	3.50	0.87	1.50	5.00
High and low fat regulation	3.47	3.50	0.97	1.00	5.00

* *Nine participants in PCA stage did not provide an age for their child but stated that they had at least one child aged 1–16 living with them. Parentheses around a component indicate that that component was not robust in the CFA for the given age group*.

The table shows that goal endorsement was similar across age groups; *stress/conflict avoidance* was the most highly endorsed goal, followed closely by *homemade food* and *shared family food*. *High & low fat regulation and occasional treats* were comparatively less strongly endorsed. All goals had a mean and median endorsement score ≥3.

## Discussion

The objectives of this study were, first, to develop a measure to capture parental goals at mealtimes and, second, to use this to describe parents' motivations when feeding children. In line with the first of these objectives, a self-report questionnaire measure was designed and tested, and found suitable for use in future research to better understand parents' goals in relation to children's eating and family mealtimes. Among a large sample of parents, 18 items describing seven distinct goals were identified. Goals include *price* and several relating to the provision of healthy food, supporting previous findings that these concepts are important in parents' decisions about feeding their children (Carnell et al., 2011; Russell et al., 2015). Due to the extensive exploratory work, our results also highlight several motivators that have not previously been considered, namely *stress/conflict avoidance, shared family food, homemade food, family involvement in mealtimes, occasional treats* and *high & low fat regulation*, confirming that parents' mealtime goals are not fully captured by measures designed for the assessment of adults' food choices.

In this study, several distinct health-related motivators emerged as individual factors, namely *homemade food, high & low fat regulation* and *occasional treats*. The separation of these factors contrasts with the approach taken in previous research, which has assumed that parents hold a global health goal, and has measured their endorsement of this (e.g., Kiefner-Burmeister et al., 2014; Hoffmann et al., 2016). The lack of a global health goal in our analysis might reflect the universality with which parents hold such a goal when planning children's meals, or at least claim to do so, given their broad awareness of the healthy eating messages promoted by healthcare providers, government and the media. It may also represent a response bias whereby parents over-estimate how much they prioritize healthy eating because they think they ought to. However, our analysis indicates that parents do vary in their endorsement of goals related to different approaches to healthy eating. The individual items within the *homemade food* factor refer to the use of raw and natural ingredients, which likely represent healthier food choices to parents (Hart et al., 2015). *High & low fat regulation* demonstrated the most variability of all the factors, indicating that some parents value low-fat food choices while others endorse high-fat choices. This variability likely reflects the complexity of the task of ensuring children have a balanced diet and a lack of clarity around health messages relating to fat, especially for children. Arguably, parents' search for balance is also reflected in the *occasional treats* goal, which describes less healthy nutritional aspirations, perhaps representing parents' desire to avoid restrictive feeding practices (Birch, 1999). These findings therefore go beyond simply stating that children's health is important to parents, and help to elucidate how parents interpret healthy feeding and eating behaviors, and the differing importance they ascribe to different health-related goals.

Our analyses also suggest that parents' goals vary somewhat according to children's age group. The majority of goals identified among the cohort as a whole remained psychometrically strong within each individual age group (i.e., *homemade food, shared family food, price, family involvement in mealtimes, stress/conflict avoidance)*. However, other goals did not. The absence of *ease of preparation* as a coherent goal for parents of younger age groups is particularly noteworthy. *Ease of preparation* aligned most closely to the factor termed “convenience” in previous research; work based on the Food Choice Questionnaire (Steptoe et al., 1995) has assumed that convenience is an important motivator for parents (Hoffmann et al., 2016). As discussed in the Introduction, “convenience” might hold different meanings among parents. We intentionally avoided using this term when labeling our component “*ease of preparation*,” as the items that most strongly loaded on this factor related very clearly to the time involved in and ease of meal preparation, rather than other items potentially falling under the heading of convenience (e.g., availability of foods to purchase). In our study, while parents of children in all age groups endorsed the individual *ease of preparation* items strongly, collectively these items do not form a coherent goal for most parents. What constitutes convenience may therefore differ for parents of children of different ages; other factors such as *stress/conflict avoidance* and *shared family food* may better represent the elements of “convenience” that matter more to parents of younger children in particular. Thus, as we suggested above in relation to “health” goals, our questionnaire may better represent the diversity of “convenience” goals that matter to parents, and the differences between parents of different age groups in the importance of these.

We also saw inconsistency between the age groups in relation to the factor *occasional treats*, perhaps because parents of secondary school-aged children are less able to monitor their children's snack consumption. The final inconsistency related to *high/low fat regulation*, likely reflecting parents' awareness of the differing nutrition advice given for children of different ages. Parents of very young children are often encouraged to provide full-fat foods, for example (NHS, 2015).

Our second stated objective was to describe parents' motivations when feeding their children. The goals endorsed in the final Family Mealtime Goals Questionnaire were explored in the combined sample of 1,140 parents. Across age groups, *stress/conflict avoidance* was the most highly endorsed goal, closely followed by *homemade food* and *shared family food*. The emphasis on *stress/conflict avoidance* is interesting; a recent study suggests that parents who try to avoid conflict at mealtimes more often concede to children's food-related demands, resulting in less healthy food choices being provided (Norman et al., 2015). If the focus on *stress/conflict avoidance* in this sample is typical of parents in general, it is possible that healthy eating interventions that contain stress-free messages may be responded to more positively. However, an unintended consequence of efforts to reduce the stress associated with mealtimes might be a decrease in the healthiness of the child's diet. Future research might explore the consequences of parents holding such potentially-conflicting goals. In terms of the goals least strongly endorsed, participants placed lower importance on *occasional treats* and *high & low fat regulation*. Nonetheless, mean and median scores for all goals were above neutral (except *high & low fat regulation*, with a median of exactly 3), indicating that all goals were endorsed by a majority of participants.

### Strengths and Limitations

The sample size in this study was large, and as such we can be confident that the FMGQ is usable with parents of younger age groups (1–11 year olds). However, our sample of parents of older children was below threshold for satisfactory CFA testing, and future work should address the suitability of the questionnaire with a larger sample of parents of this age group.

In addition, despite the large sample size in our study, participants were predominantly well-educated and employed. Parents from other socio-demographic groups might report different feeding goals, or display more variability in their prioritization of these. Likewise, we did not seek to recruit parents of children with feeding or eating disorders. The lack of a global health goal or broader convenience goal might, therefore, be specific to our sample. In this study, several global health-related items were dropped because they demonstrated little between-subject variability; although three items (heavily skewed but otherwise psychometrically strong) were carried into the CFA stage to ensure this concept was represented, these remained skewed and their inclusion worsened the model's fit. However, we provide these items in Appendix III for transparency and to enable their inclusion in future investigations of health goals among other populations.

Strictly, guidelines around questionnaire and scale development recommend that no single component should have fewer than three items that load on it Hair et al. (2010). Our questionnaire has three components including only two items, which arguably reduces the reliability of these factors. However, given our previous findings around parents' low tolerance of long questionnaires (Snuggs et al., 2016) and the psychometric strength of these components in the PCA, we gave priority to keeping the final questionnaire brief.

It is worth noting that some components and items that were eliminated due to statistical weakness may be important to some parents. For example, in the preliminary item generation work, some parents expressed the view that their priority was to “get food into their child”; responses to open-ended questions illustrated their frustration with ensuring sufficient energy consumption, and the lesser importance of the food's nutritional content (“Getting them to eat something so they're no longer hungry. If it's healthy then that's good.”) This goal did not meet threshold for inclusion in the final questionnaire; nor did “prevention of fussy eating” or “portion control,” both of which were highlighted by some parents at the item generation stage. While it is important that the methodological rigor of measure development does not come at the expense of losing valid indices of the construct in question, it is also true that, for a questionnaire to be useable, it cannot measure everything. The FMGQ allows the measurement of the principle mealtime goals that parents have expressed and on which they show individual differences. However, we acknowledge that our measure, although practical and reliable, may not capture the full complexity of parents' motivations around mealtimes.

The aim of this study was to examine the extent to which parents endorsed different mealtime-related goals and to develop an instrument that would discriminate between parents in terms of the goals that are important to them. Future work could usefully consider the influence of the different goals on parents' behavior. For example, parents could be asked to rate the goals in order of importance, in terms of the extent to which each influences their choices about what to prepare for their child's meals. They might also be asked to identify any goals that they perceive as conflicting with one another, causing difficulty when making decisions about mealtimes. Development of the tool to capture parents' priorities in this way would enable identification of any changes in these over time, such as with age, or treatment. With this approach, it might also be useful to reconsider some of the items dropped in the preliminary stages of the scale development (Appendix I). As suggested above, some of the items dropped in this study may be more meaningful for other population groups (e.g., parents of children with obesity or eating disorders).

### Implications for Research and Practice

In addition to enabling the measurement of parents' feeding goals, the Family Mealtime Goals Questionnaire (FMGQ) could support the design of interventions to change feeding practices. To date, interventions have assumed that parents prioritize health goals when making decisions about children's meals. This might reflect a bias in the underpinning research; studies of family eating typically rely on self-selecting participants, in which parents who are motivated to provide healthy foods may be over-represented. To our knowledge, no intervention study has incorporated parents' broad range of feeding goals in their design. The few that include “goal-setting” as a component have typically required participants to select among prescribed health-related goals (e.g., Draxten et al., 2016). The FMGQ does not assume that health is parents' principal motivation. It facilitates a more sophisticated understanding of parents' feeding goals, allowing the development of interventions that better align with parents' motivations. For example, among parents for whom a low-cost goal is priority, interventions focusing on inexpensive ways to create healthy meals may be successful. Similarly, parents who prioritize shared family mealtimes may benefit most from support with planning healthy meals that appeal to a range of tastes and ages.

Where a child's eating is disordered, insight into parents' mealtime priorities and any areas of conflict between these may facilitate care coordination between dieticians and psychologists. The measure could also be used to investigate and address the potential discrepancy between parents' feeding goals and practices and the relationship between parental goals and children's eating behaviors. For example, parental feeding goals may be linked to cooking self-efficacy, which has been shown to be associated with increased adherence to nutritional guidelines (Arcan et al., 2019). Given the importance of *stress/conflict avoidance* at family mealtimes it would also be of interest to examine whether there is any association between this goal and family meal frequency, thought to be a protective factor against family conflict (Hammons and Fiese, 2011).

Our results clearly indicate that individuals can endorse several feeding goals simultaneously. Less clear is whether this leads to perceived goal conflict, hindering the achievement of their goals (Emmons and King, 1988; Boudreaux and Ozer, 2013). For example, if a parent has goals of both *stress/conflict-avoidance* and *homemade food* but perceives preparing home-made meals to be stressful, one goal is likely to suffer. Goal conflict can be addressed by goal facilitation (Presseau et al., 2010; Boudreaux and Ozer, 2013); when designing interventions, healthcare practitioners could facilitate the achievement of multiple goals to reduce conflict, leading to healthier, happier mealtimes. Future research should investigate whether conflict is common in relation to mealtime goals and whether support in reducing such conflict enhances feeding practices or mealtime characteristics.

## Data Availability

The datasets generated for this study are available on request to the corresponding author.

## Author Contributions

SS, CH-P, and KH were involved in the conception and design of the study and in interpretation and writing up of results. SS was responsible for data collection and data analysis.

### Conflict of Interest Statement

The authors declare that the research was conducted in the absence of any commercial or financial relationships that could be construed as a potential conflict of interest.

## Footnotes

^1^www.getfeedback.com
