# Supplementary material for: Development of a Parental Feeding Goal Measure: The Family Mealtime Goals Questionnaire
Source: Front Psychol. 2019 Mar 12;10:455. doi: 10.3389/fpsyg.2019.00455 (PMC6422867; doi:10.3389/fpsyg.2019.00455)
Supplement: Supplementary file 1 [file Table_1.docx]

**Appendix I: Pool of 66 items before discards in Principal Components Analysis stage**

| I don’t want to spend a long time preparing food for my child |
| --- |
| I don't want my child to be a fussy eater |
| I don't want to get stressed thinking about mealtimes |
| I don't want to give my child fatty foods |
| I don't want to give my child processed foods |
| I don't want to prepare different foods for different family members |
| I don't want to waste food |
| I want mealtimes to be an opportunity for my child to chat to me |
| I want mealtimes to be an opportunity for my family to be together |
| I want my child and I to eat the same food |
| I want my child to appreciate different types of foods* |
| I want my child to be free to eat unhealthy food sometimes |
| I want my child to behave well at mealtimes* |
| I want my child to eat a reasonable amount of food at every meal |
| I want my child to eat all the food I give them at a meal |
| I want my child to eat high energy food more than highly nutritious food |
| I want my child to eat something at mealtimes, regardless of what it is |
| I want my child to enjoy healthy food* |
| I want my child to enjoy their food* |
| I want my child to have a healthy diet overall* |
| I want my child to have enough food to keep them going for the day |
| I want my child to learn about different foods* |
| I want my child to like the taste of the food* |
| I want my child to look forward to mealtimes* |
| I want my child to make sensible choices about their food* |
| I want my child to think the food looks appealing |
| I want my child to understand the difference between healthy and unhealthy foods* |
| I want my child’s meal to include protein, carbohydrates and fruit/vegetables* |
| I want the whole family to help out with mealtimes |
| I want to avoid arguments at mealtimes |
| I want to avoid mealtimes being stressful* |
| I want to avoid mess at mealtimes |
| I want to be organised about my child’s meals |
| I want to choose food for my child that is easy for me to prepare |
| I want to choose food that my child can help prepare |
| I want to control my child’s weight |
| I want to enjoy preparing food for my child |
| I want to ensure my child has a balanced diet overall* |
| I want to get my child involved with things like setting the table or clearing up |
| I want to give my child enough fruit and veg* |
| I want to give my child enough variety* |
| I want to give my child food that is high in energy |
| I want to give my child food that is low in fat |
| I want to give my child food that is low in sugar |
| I want to give my child food that is nutritious* |
| I want to give my child food that will fill them up |
| I want to give my child food they like, to avoid a fuss |
| I want to give my child fresh food* |
| I want to give my child good quality food* |
| I want to give my child healthy food* |
| I want to give my child home-cooked food |
| I want to give my child sugary treats sometimes |
| I want to give my child the right portion size |
| I want to introduce my child to foods they haven't tasted before* |
| I want to keep costs down |
| I want to keep to my budget |
| I want to make sure I don’t lose my temper at mealtimes |
| I want to make sure my child eats something at mealtimes, even if they’re not hungry |
| I want to make sure my child is never hungry |
| I want to offer my child an alternative if they don't want the food I give them |
| I want to offer my child foods with different textures |
| I want to prepare food for my child using natural ingredients |
| I want to prepare food for my child using raw ingredients |
| I want to prepare food that all my family will eat |
| I want to prepare food that my child will eat |
| I want to prepare food that my child will like* |

*Indicates dropped from the model due to severely skewed distribution. All other discards due to unsatisfactory factor loading.

**Appendix II: Figures 2 – 4, showing Confirmatory Factor Analysis models for different age groups**

**Figure 2: Summary of Confirmatory Factor Analysis model for parents of pre-schoolers**

**Figure 3: Summary of Confirmatory Factor Analysis model for parents of primary school aged children**

**Figure 4: Summary of Confirmatory Factor Analysis model for parents of secondary school aged children**

*
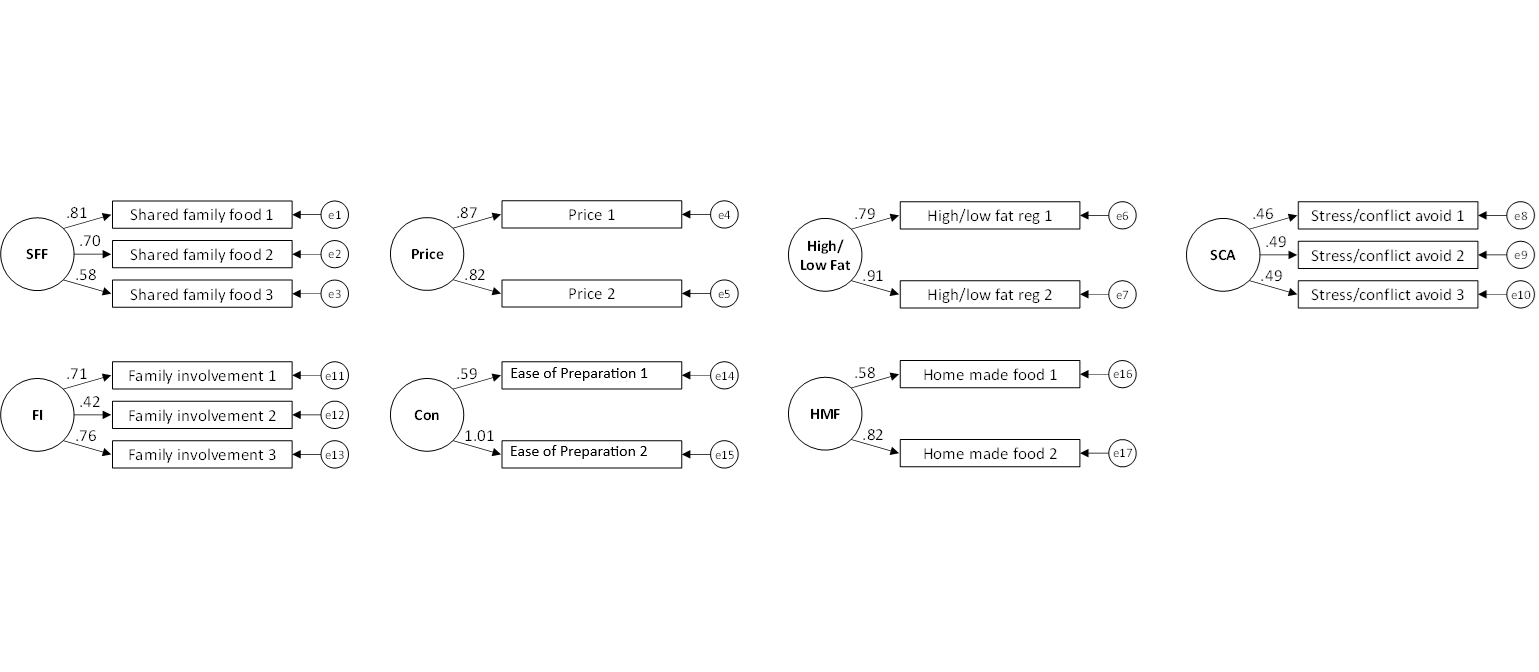
*

**Appendix III: The final Family Mealtime Goals Questionnaire**

Thinking about your child’s mealtimes, how strongly do you agree with the following statements? [*Strongly agree = 5, Agree = 4, Neither agree nor disagree = 3, Disagree = 2, Strongly disagree = 1*]

| *Component 1: Shared family food* |
| --- |
| 1 - I don't want to prepare different foods for different family members |
| 2 - I want my child and I to eat the same food |
| 3 - I want to prepare food that all my family will eat |
| *Component 2: Stress/conflict avoidance* |
| 4 - I want to avoid arguments at mealtimes |
| 5 - I don't want to get stressed thinking about mealtimes |
| 6 -I want to make sure I don’t lose my temper at mealtimes |
| *Component 3: Homemade food* |
| 7 - I want to prepare food for my child using natural ingredients |
| 8 - I want to prepare food for my child using raw ingredients |
| 9 - I want to give my child home-cooked food |
| *Component 4: Family involvement in mealtimes* |
| 10 - I want the whole family to help out with mealtimes |
| 11 - I want to choose food that my child can help prepare |
| 12 -I want to get my child involved with things like setting the table or clearing up |
| *Component 5: Price* |
| 13 - I want to keep to my budget |
| 14 - I want to keep costs down |
| *Component 6: Occasional treats* |
| 15 - I want to give my child sugary treats sometimes |
| 16 - I want my child to be free to eat unhealthy food sometimes |
| *Component 7: High and low fat regulation* |
| 17 - I want to give my child food that is low in fat |
| 18 - I don't want to give my child fatty foods |

Because of the notable absence of a global ‘health’ factor in the PCA (which we attribute to the fact that health-related items were dropped, as exclusively endorsed by parents), we reintroduced the three psychometrically-strongest health-related items in Step 3. These items were similarly skewed in the CFA and did not improve the model, and are therefore not reported in detail in the full text. The items can, however, be seen in Component 9, below.

| *(Optional component 8: Ease of preparation)* |
| --- |
| 19 - I want to choose food for my child that is easy for me to prepare |
| 20 - I don’t want to spend a long time preparing food for my child |
| *(Optional component 9: Health)* |
| 21. I want to give my child enough fruit and veg |
| 22. I want to ensure my child has a balanced diet overall |
| 23. I want to give my child food that is nutritious |

Scores for each subscale are calculated by diving the sum of all items by the number of items.

E.g. Component 1 score = (Item 1 score + Item 2 score + Item 3 score)/3.
